# What are the clinical (endoscopic) differentials of celiac disease in dyspeptic syndrome?

**DOI:** 10.1590/0102-67202025000018e1887

**Published:** 2025-07-21

**Authors:** Manoela Aguiar CRUZ, Nicolau Gregori CZECZKO, Leticia Elizabeth Augustin Czeczko RUTZ, Matheus Toniolo MALAFAIA

**Affiliations:** 1 Faculdade Evangélica Mackenzie do Paraná, Medical Research Institute – Curitiba (PR), Brazil.

**Keywords:** Dyspepsia, Celiac Disease, Glutens, Prevalence, Dispepsia, Doença Celíaca, Glútens, Prevalência

## Abstract

**Background:**

Dyspepsia is a set of symptoms of the upper abdomen and has a prevalence of 10–45% of the population with different etiological possibilities, including celiac disease.

**Aim:**

The aim of this study was to evaluate the prevalence of celiac disease in patients with a clinical diagnosis of dyspeptic syndrome.

**Methods:**

This is an observational research, based on a review of medical records of patients treated for uninvestigated dyspepsia. Patients over 18 years of age, with uninvestigated dyspepsia, and who had upper endoscopy, total immunoglobulin A (IgA), and antitissue transglutaminase IgA were included. Those with diarrhea, constipation, malabsorption, refractory lactose intolerance, or who presented extraintestinal signs or symptoms suggestive of celiac disease were excluded.

**Results:**

The initial sample was 1,802 records and the final 200 patients. Considering the total sample, the average age was 45.13 years and the female sex was predominant. Symptoms associated with gluten were reported in 6% of the patients. The antitissue transglutaminase IgA was positive in 1.5% of the patients. Considering the sample of 100 patients, the diagnosis of celiac disease had a prevalence of 3%.

**Conclusions:**

The prevalence of celiac disease in patients with a clinical diagnosis of dyspeptic syndrome was 3%.

## ARTICLE HIGHLIGHTS

- Celiac disease is autoimmune disease associated with gluten that affects the small intestine
- Dyspeptic symptoms may be part of the clinical manifestations of celiac disease
- The prevalence of celiac disease in patients with a clinical diagnosis of dyspeptic syndrome was 3%

## CENTRAL MESSAGE

Celiac disease is an autoimmune disease associated with gluten that affects the small intestine. Dyspeptic symptoms may be part of the clinical manifestations of celiac disease, but it is not clear whether patients with dyspepsia alone have a higher risk of celiac disease than individuals in the general population. Therefore, there are discrepancies in relation to the prevalence of celiac disease in dyspeptic syndrome, and hence further epidemiological studies are necessary in order to establish in the future in whom celiac disease research should be performed.

## PERSPECTIVES

The prevalence of celiac disease in patients with a clinical diagnosis of dyspeptic syndrome was 3%, and the sample was characterized by an average age of 45.13 years; predominance of females; and glutenrelated symptoms in 6%; endoscopic signs of celiac disease in 2.5%; serological positivity of 1.5%; and histological signs of celiac disease in 3% of the biopsied patients.

## VISUAL ABSTRACT

**Figure:**
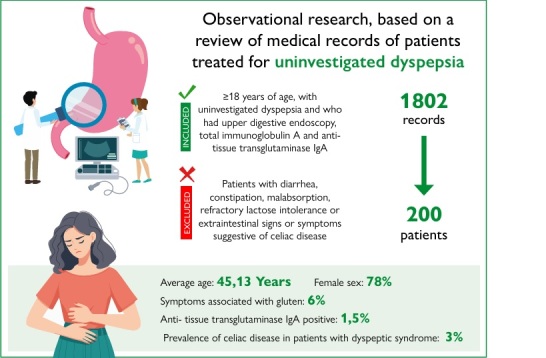

## RESUMO

**Racional:**

A dispepsia é um conjunto de sintomas do abdome superior e apresenta prevalência de 10-45% da população, cursando com diversas possibilidades etiológicas, incluindo a doença celíaca.

**Objetivos:**

Avaliar a prevalência de doença celíaca em pacientes com diagnóstico clínico de síndrome dispéptica.

**Métodos:**

Estudo observacional, baseado na revisão de prontuários de pacientes atendidos por dispepsia não investigada. Foram incluídos pacientes acima de 18 anos, com essa dispepsia e que possuíssem endoscopia, imunoglobulina A total e antitransglutaminase IgA. Foram excluídos os com quadro diarreico, constipação, má-absorção, intolerância à lactose refratária ou que apresentassem sinais ou sintomas extraintestinais sugestivos de doença celíaca.

**Resultados:**

A amostra inicial foi de 1.802 registros e a final de 200 pacientes. Considerando o total da amostra a idade média foi de 45,13 anos e o sexo feminino foi predominante. Os sintomas associados ao glúten foram relatados em 6%. O anticorpo antitransglutaminase foi positivo em 1,5%. Considerando a amostra de 100 pacientes, o diagnóstico de doença celíaca teve prevalência de 3%.

**Conclusões:**

A prevalência de doença celíaca em pacientes com diagnóstico clínico de síndrome dispéptica foi de 3%.

## INTRODUCTION

 Dyspepsia is a group of upper abdominal symptoms that affects approximately 10–45% of the world’s population and is characterized by epigastric pain or discomfort, epigastric burning, early satiety, and postprandial fullness^29,44^.Celiac disease is an autoimmune disease associated with gluten that affects the small intestine^5,17,30^. 

 Dyspeptic symptoms may be part of the clinical manifestations of celiac disease, but it is not clear whether patients with dyspepsia alone have a higher risk of celiac disease than individuals in the general population^3^. This doubt has an impact on the indication of celiac disease research in this scenario. In view of the above, and considering that there are discrepancies in relation to the prevalence of celiac disease in dyspeptic syndrome, further epidemiological studies are necessary in order to establish in the future in whom celiac disease research should be performed.

 The objectives of this study were to evaluate the prevalence of celiac disease in patients with a clinical diagnosis of dyspeptic syndrome and study the epidemiology of the sample, symptoms, endoscopic outcome, histological findings, and serological results. 

## METHODS

 This is an observational prevalence study, based on a review of the medical records of patients treated for dyspepsia that was not investigated and received a favorable opinion from the Research Ethics Committee of Faculdade Evangélica Mackenzie do Paraná under number 5.123.320 on November 24, 2021 (CAAE 53310321.3.0000.0103). 

 Dyspepsia was defined as epigastric pain or discomfort, epigastric burning, early satiety, and postprandial fullness, which may or may not include several symptoms such as belching, nausea, vomiting, heartburn, regurgitation, anorexia, and distension in the upper abdomen. Uninvestigated dyspepsia was when, until the initial evaluation, there was no investigation to reveal the underlying diagnosis. 

 Data collection took place at a private gastroenterology clinic in Curitiba (PR), which began in December 2021 and ended in February 2022. The collection method was through the issuance of a report referring to all records of attendance from November 2020 to November 2021. The initial sample consisted of 1802 records of attendance. The medical records of each patient were accessed for inclusion in the sample. Of these, 990 had dyspeptic complaints, totaling 441 patients, as there were patients with more than one visit. 

 The inclusion criteria were patients treated for dyspepsia that was not investigated; age over 18 years; having undergone upper digestive endoscopy (UDE) with a description of the second duodenal portion and investigation of *Helicobacter pylori* by the urease and/or anatomopathological method; having performed total immunoglobulin A (IgA) measurement and collection of antitissue transglutaminase IgA; and those who had tests performed during a gluten-rich diet. 

 The exclusion criteria were presence of concomitant diarrhea or constipation; presence of malabsorption, considered as a report of weight loss, iron deficiency, and B12 or folic acid deficiency report by the patient or evidenced in previous laboratory tests brought in the first consultation; refractory lactose intolerance, considered as the persistence of signs and symptoms in an indivudual with a previous diagnosis of lactose intolerance correctly performing the diet and the use of the lactase enzyme; and presence of extraintestinal signs or symptoms suggestive of celiac disease, such as dermatitis herpetiformis, amenorrhea, infertility, secondary hyperparathyroidism, anemia, liver-enzyme abnormalities, osteoporosis, headache, ataxia, and peripheral neuropathy. 

 After applying the inclusion and exclusion criteria and removing duplicate patients, the final sample resulted in 200 patients. Of these, 100 patients had antibody dosage, upper gastrointestinal endoscopy, and duodenal histology. The other 100 patients had antibody measurements and upper gastrointestinal endoscopy. 

 Sex, minimun age, maximum age, and mean age were evaluetad. The following were considered as disease-related autoimmune comorbidities: Type 1 diabetes mellitus, autoimmune thyroid diseases, autoimmune polyglandular syndrome Type III (autoimmune thyroiditis and immune-related diabetes), and atopic dermatitis. The previous existence of celiac disease in the first-degree family, previously diagnosed lactose intolerance, and dyspeptic symptoms associated with gluten were considered according to the report in the anamnesis. 

 The results of UDE were classified according to the following findings: presence of *H. pylori* (evaluated by histology with Giemsa stain or urease method), presence of peptic ulcer, presence of gastric cancer, and a duodenal appearance at upper gastrointestinal endoscopy (atrophy, reduction of folds, mucosal irregularity or nodularity, fissures, or mosaic appearance) and duodenal biopsy. 

 Duodenal histology was performed in 100 patients, and its study was done with hematoxylin-eosin staining and interpreted by a pathologist. The result was described as normal or consistent with celiac disease. To this end, the Marsh-Oberhuber classification was used and celiac disease was considered when consistent with types 2 and 3. 

 The total IgA level was considered normal or reduced according to the collected laboratory reference value. The result of the antitissue transglutaminase IgA measurement was also considered negative or positive according to the collected laboratory reference value. 

 The celiac disease diagnosis was established when there was a positive antitissue transglutaminase IgA with normal total IgA and duodenal histology findings were consistent with Marsh-Oberhuber type 2 or 3. 

 Data were collected and plotted in an Excel table. All data collected and stored have been encrypted in order to preserve the identity of the participant. The results were described as numerical absolute and percentage results. Quantitative variables were described as mean, minimum, and maximum values. The prevalence of positive serology for celiac disease was calculated, as well as the prevalence of celiac disease diagnosis within the sample studied. The confidence interval (CI) was 95%, and the Clopper-Pearson approach was used to calculate it. 

## RESULTS

 Of the 200 patients in the sample, 156 were women (78%) and 44 were men (22%). Their age ranged from 18 to 83 years, with a mean of 45.13 years. Of the 200 patients, 23 (11.5%) reported having an associated comorbidity, hypothyroidism in all cases; five (2.5%) had first-degree relatives with celiac disease; 58 (29%) had been previously diagnosed with lactose intolerance; and 12 (6%) had gluten-related dyspeptic symptoms. 

 The UDE was considered normal in 44 patients (22%) and in 30 (15%), there was *H. pylori infection*. In one case (0.5%), there was a diagnosis of peptic ulcer and in another (0.5%) gastric cancer. Regarding the duodenal appearance, five patients (2.5%) had an aspect suggestive of celiac disease, such as atrophy, reduction of folds, irregularity, mucosal nodularity, fissures, or mosaic appearance. A total of 150 endoscopies (75%) had other findings with no impact on the diagnosis. These findings do not add up to 100% of the cases because the same patient could present more than one alteration. 

**Figure 1:**
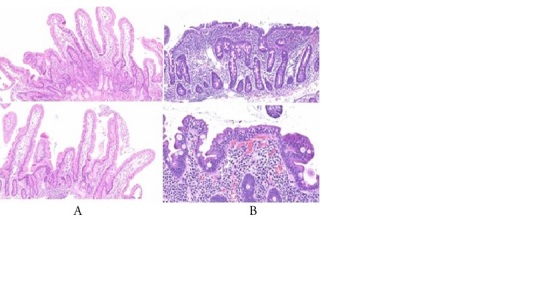
Histology of the duodenum stained with hematoxylin-eosin: (A) normal, with the presence of high, slender villi, in a ratio equal to or greater than 3:1 in relation to the crypts; absence of intraepithelial lymphocytosis; (B) compatible with celiac disease, with the presence of villous atrophy, crypt hyperplasia, and intraepithelial lymphocytosis.

 One-half of the sample (n=100) underwent duodenal biopsy and histological analysis to investigate celiac disease. Of these, three had findings compatible with Marsh-Oberhuber types 2 and 3 (Figure 1B). The others had duodenal histology with no findings suggestive of celiac disease (Figure 1A). 

 All patients had normal total IgA levels. Therefore, none of them were IgA deficient. Of the total of 200 participants, three had a positive antitissue transglutaminase IgA, resulting in an estimated prevalence of 1.5% (95%CI given by 0.3–4.3%). 

 Of the 100 patients who underwent biopsies, three had a diagnosis of celiac disease, which was established by positive serology and histology. This result has an estimated prevalence of 3% (95%CI given by 0.6–8.5%). 

## DISCUSSION

 There was a predominance of females (78%) and a mean age of 45.13 years, consistent with the literature for dyspepsia^4,25,44^. Among the autoimmune diseases associated with celiac disease, thyroid disorders are the most commonly found23. In the sample, the only comorbidity associated with celiac disease was hypothyroidism. This comorbidity was reported by 23 patients (11.5%), one of whom was diagnosed with celiac disease. 

 The medical literature demonstrates that there is an increased risk of celiac disease in the first-degree relatives of celiac individuals^41^. The present study reported five patients with such a family relationship (2.5%), but there was no positive association between celiac diagnosis and a family history. However, Behforouz et al.^4^ identified three patients in a sample of 530 dyspeptic individuals studied for celiac disease, who had a greater association between celiac disease and a positive family history in the first-degree relatives. 

 Lactose intolerance was reported in 29% of the sample. This prevalence is lower than that found in a Brazilian study, which reported it at 44.11% in the south region, a value that includes patients with clinical manifestations other than dyspepsia, making it impossible to directly compare the results^35^. Global references indicate that the prevalence of lactose intolerance around the world is uncertain due to its regional variables and different testing methodologies. Despite this, the presence of lactose maldigestion is believed to be around 68% worldwide^28^. In the sample, among the three celiacs, only one had reported lactose intolerance. There are no data regarding the association of celiac disease and lactose intolerance in patients with dyspepsia not investigated for comparison. 

 A total of 12 patients reported worsening of dyspeptic symptoms with gluten intake; of these, the disease was confirmed in one patient. This fact can be justified by two possibilities: the presence of celiac disease with dyspeptic symptoms (n=1), or the presence of non-celiac gluten sensitivity (n=11), characterized by the presence of symptoms with gluten ingestion, without the presence of antibodies and without histological signs^21,31^. 

 The evaluation of the presence of autoimmune comorbidity, first-degree relatives affected by the disease, lactose intolerance, and gluten-related symptoms was an attempt to find a positive association with celiac disease. It would be a way of evaluating the individuals who would be at higher risk in the anamnesis. However, due to the small number of celiacs found, such an association could not be made. 

*H. pylori* infection was found in 15% of the patients, a rate lower than the general Brazilian population, according to studies by Coelho et al.^6^ with 70% and Toscano et al.^45^ with 35.4%. Its prevalence was also lower than expected for Latin America, which is around 57.6% globally and 69.2% in adults. The difference in the prevalence between the present study and the literature may be related to the fact that there is a higher prevalence of *H. pylori* in Brazilian public services (42%) than in private healthcare (25.6%)^6,27^. The absence of organic causes for dyspepsia such as *H. pylori* infection, gastric cancer, peptic ulcer disease, and celiac disease corresponded to more than 80% of the sample. This result was slightly higher than that found by Harer and Hasler^11^, who reported that 70% of dyspepsia not investigated had a negative endoscopic study and that 50–60% would be classified as functional dyspepsia. Despite this, the result is comparable to that expected in the literature because according to Ford et al.^10^ less than 20% of the dyspepsia not investigated are due to organic diseases and about 80% of the individuals will be classified as having functional dyspepsia. 

 Regarding the duodenal appearance in upper gastrointestinal endoscopy, five patients showed alterations suggestive of celiac disease. However, only one patient had histology findings suggestive of celiac disease and positive antibodies, confirming the diagnosis of celiac disease. The others were both histology and serology negative, ruling out the diagnosis. Lecleire et al.^20^ stated that endoscopic markers are not reliable for the detection of celiac disease in dyspeptic individuals, making it difficult to define who to biopsy. Despite this, Behforouz et al.^4^ reported that routine biopsy of the duodenum of dyspeptic individuals is not the best way to investigate celiac disease in dyspeptic patients, but rather individualizing it according to the results of serology, complete history, and careful endoscopic evaluation. Another factor to be considered that can help in the endoscopic detection of villus atrophy is the use of an endoscope with image magnification, as it has greater sensitivity in detecting atrophy compared to endoscopy with high-definition white light^36^. 

 In this sample, 50% of the patients (n=100) were biopsied for histological analysis of the duodenum. The criterion used to define the biopsy was the request of the attending physician or the identification of endoscopic signs. In clinical practice, several factors may impact this decision, such as the clinical suspicion of organic disease, ease of access to endoscopic examination in Brazil, and/or the opportunity to perform the biopsy during the endoscopy already requested, leading the physician to ask the endoscopist to perform the duodenal biopsy regardless of the macroscopic aspect. Another factor is the moment of endoscopy, in which the patient may present some alteration, whether peptic or atrophic, leading the endoscopist to perform the biopsy. Such individualized conduct is in accordance with the study by Behforouz et al.^4^ which, as previously mentioned, advocates a personalized approach to define which patient should be biopsied. 

 Of these 100 patients who underwent biopsies, three had histology compatible with celiac disease. Of these, two had a normal duodenal appearance, which is in agreement with studies by Bardella et al.^3^ and Leclaire et al.^20^, who state that endoscopic markers are not reliable for the detection of celiac disease in dyspeptic individuals. 

 According to Husby et al.^12^, Penny et al.^34^, and RubioTapia et al.^38^, the total IgA dosage should accompany celiac serology in order to avoid false negatives in the case of IgA deficiency. This condition is prevalent in 1–400 to 1–800 individuals in the general population and more common in celiacs, 2–3 out of every 100 individuals^23,39^. In our sample, all patients (n=200) had total IgA levels and all patients had normal laboratory limits. 

 Considering the total of 200 patients, the prevalence of antitissue transglutaminase IgA positivity in the present study was 1.5%. The calculated CI estimated that there was a 95% chance that the 0.3%–4.3% range contained the true prevalence of positive antibody results in the target population. 

 The prevalence of antitissue transglutaminase IgA positivity in the general world population and in the Brazilian population is around 1.4–1.5%^18,32,42^. In the context of dyspepsia, worldwide studies indicate a serological prevalence higher than that of the general population. The study by Behforouz et al.^4^ found a 6.8% prevalence of antibody positivity. Similar results were found by Ford et al.^9^, Nejad et al.^31^, Roshanzamir et al.^37^, and Keshavarz et al.^14^, who obtained a prevalence of 7.9, 8, 8.6, and 7%, respectively. An Indian study^40^ showed lower values, of 5%, close to the result of a systematic review and metaanalysis by Singh et al.^43^ which identified 4.8% positivity. 

 In contrast to these international results, a 2012 Brazilian study conducted by Machado et al.^24^ evaluated 399 dyspeptic patients and found 1.3% celiac serology positivity, with a predominance in women and a mean age of 49.6 years. The serological prevalence of the present study (1.5%) is lower than that found in the medical literature investigating celiac disease in dyspepsia but very similar to the Brazilian study by Machado et al.^24^ This suggests the possibility that the Brazilian seroprevalence is lower than the world seroprevalence in the context of dyspepsia. 

 The prevalence of celiac disease in the world population is 0,7%, being lower in the Brazilian population, which is estimated to be between 0.3 and 0.4%^1,18,26,32,42^. In the medical literature, the results are conflicting when studying celiac disease in dyspepsia. A meta-analysis of a 2022 systematic review reports that there is a lot of heterogeneity and a moderate risk of bias in the literature on the subject^43^. 

 Considering that, according to Kelly^13^, the diagnosis of celiac disease requires a combination of antibody positivity, normal total IgA, and histology compatible with MarshOberhuber type 2 or 3, and our study was able to evaluate the prevalence of celiac disease in 100 patients. Its prevalence in the dyspeptic syndrome found in the present study was 3%. The calculated CI estimates that there is a 95% chance that the 0.6–8.5% range contains the true prevalence in the target population. This result refers to half of our sample (100 patients) since the analysis of serology and duodenal histology is necessary for such a diagnosis. The value of 3% is higher than expected for the world population and for the healthy Brazilian blood donor population^1,26,32^. 

 Our results were similar to those found in other studies on the prevalence of celiac disease in dyspepsia. Behforouz et al.^4^, Ford et al.^9^, Nejad et al.^31^, and Roshanzamir et al.^37^ found 2.8, 3.2, 2.5, and 3.7% of it, respectively, suggesting values above the expected for the population. That is, that the prevalence of celiac disease in dyspeptic individuals would be higher than in relation to the general population. However, there are also inferior results, such as those obtained by Lasa et al.^16^, Dekante et al.^8^, Altintaş et al.^2^, Sharma et al.^40^, Özaslan et al.^33^, and Singh et al.^43^, who found a prevalence of celiac disease in dyspepsia of 1.25, 0.9, 1.44, 1.1, 1.5, and 1.5%, respectively, values close to those expected for the general population. Two studies in the Brazilian literature^22,24^ found results similar to the studies mentioned above, 1.4% and 0.75%, respectively. Although these values are close to those expected for the general world population, they are also values above what is expected for the healthy Brazilian population. 

 In the world literature, seronegative celiac disease has a prevalence of 2–15% of celiacs. It is characterized by being a special situation in which the antibody dosage is negative, but there is an alteration in duodenal histology and genetic positivity compatible with celiac disease^19^. This condition can be evaluated in the group of 100 patients who had histology and serology. No patient with this diagnosis was found, possibly due to the low number of biopsied cases. A Brazilian study conducted by Kotze et al.^15^ in 2020 addresses seronegative celiac disease. The study reinforces the importance of following the investigation with duodenal histology in patients with compatible symptoms and a family history, even with negative antibodies. 

 Finally, a review study on celiac disease in dyspeptic syndrome reinforces the individualized approach, especially in the context of a family history, gluten-related symptoms, and autoimmune comorbidities^7^. 

## CONCLUSIONS

 The prevalence of celiac disease in patients with a clinical diagnosis of dyspeptic syndrome was 3%, and the sample was characterized by an average age of 45.13 years; predominance of females; and gluten-related symptoms in 6%; endoscopic signs of celiac disease in 2.5%; serological positivity of 1.5%; and histological signs of celiac disease in 3% of the biopsied patients. 

## Data Availability

The datasets generated and/or analyzed during the current study are available from the corresponding author upon reasonable request.
